# Neuroanatomical Correlates of Recognizing Face Expressions in Mild Stages of Alzheimer’s Disease

**DOI:** 10.1371/journal.pone.0143586

**Published:** 2015-12-16

**Authors:** Laurie-Anne Sapey-Triomphe, Rolf A. Heckemann, Nawele Boublay, Jean-Michel Dorey, Marie-Anne Hénaff, Isabelle Rouch, Catherine Padovan, Alexander Hammers, Pierre Krolak-Salmon, Alzheimer’s Disease Neuroimaging Initiative

**Affiliations:** 1 The Neurodis Foundation, CERMEP Imagerie du Vivant, Lyon, France; 2 Brain Dynamics and Cognition, Lyon Neuroscience Research Center, INSERM U1028, CNRS UMR 5292, Lyon, France; 3 Ecole Normale Supérieure de Lyon, Lyon, France; 4 MedTech West at Sahlgrenska University Hospital, University of Gothenburg, Gothenburg, Sweden; 5 Division of Brain Sciences, Imperial College London, London, United Kingdom; 6 Clinical and Research Memory Center of Lyon, Hôpital des Charpennes, Hospices Civils de Lyon, Lyon, France; 7 Department of Medical Information and Research Evaluation, Hospices Civils de Lyon, Lyon, France; 8 University Lyon 1, F-69000, Lyon, France; 9 Centre Hospitalier Le Vinatier, Pôle Est, Bron, France; 10 Division of Imaging Sciences and Biomedical Engineering, King’s College London, London, United Kingdom; University of Manchester, UNITED KINGDOM

## Abstract

Early Alzheimer’s disease can involve social disinvestment, possibly as a consequence of impairment of nonverbal communication skills. This study explores whether patients with Alzheimer’s disease at the mild cognitive impairment or mild dementia stage have impaired recognition of emotions in facial expressions, and describes neuroanatomical correlates of emotion processing impairment. As part of the ongoing PACO study (personality, Alzheimer’s disease and behaviour), 39 patients with Alzheimer’s disease at the mild cognitive impairment or mild dementia stage and 39 matched controls completed tests involving discrimination of four basic emotions—happiness, fear, anger, and disgust—on photographs of faces. In patients, automatic volumetry of 83 brain regions was performed on structural magnetic resonance images using MAPER (multi-atlas propagation with enhanced registration). From the literature, we identified for each of the four basic emotions one brain region thought to be primarily associated with the function of recognizing that emotion. We hypothesized that the volume of each of these regions would be correlated with subjects’ performance in recognizing the associated emotion. Patients showed deficits of basic emotion recognition, and these impairments were correlated with the volumes of the expected regions of interest. Unexpectedly, most of these correlations were negative: better emotional facial recognition was associated with lower brain volume. In particular, recognition of fear was negatively correlated with the volume of amygdala, disgust with pallidum, and happiness with fusiform gyrus. Recognition impairment in mild stages of Alzheimer’s disease for a given emotion was thus associated with less visible atrophy of functionally responsible brain structures within the patient group. Possible explanations for this counterintuitive result include neuroinflammation, regional β-amyloid deposition, or transient overcompensation during early stages of Alzheimer’s disease.

## Introduction

Interpersonal communication relies on verbal conversation, but also on non-verbal cues such as facial emotional expressions. Interpreting facial expressions is crucial to recognition of the mental state of others and to successful social interaction. In Alzheimer’s disease (AD), these skills are impaired [1–3], and this impairment can lead to compromised communication and increased caregiver burden. AD is characterized at the early stages by an episodic memory decline, but other cognitive domains, like executive function and social cognition, may also be impaired [3, 4].

Previous work yielded inconsistent results concerning AD patients’ abilities to recognize emotions on faces. Most of the relevant studies report specific impairments for sadness, anger and fear [2, 5, 6]. However, only a few studies [3, 7–10] investigated this ability at early stages corresponding to mild cognitive impairment (MCI) or prodromal and mild dementia stages of AD. MCI is a transitional state between normal ageing and dementia, where patients present some deficits of cognition with relatively preserved activities of daily living and autonomy [11]. AD at the MCI or mild dementia stage is characterized by episodic memory impairment, cognitive decline to various degrees, and entorhinal cortex and hippocampal atrophy [12], as well as amygdala atrophy [13, 14]. As early atrophy occurs in brain regions crucial for emotion processing, it might be linked to social disinvestment and behavioural symptoms, early social cognition impairment, and emotion recognition impairment.

Emotions can be classified into three categories: basic emotions (happiness, surprise, sadness, fear, anger, and disgust), motivational states, and social emotions [15]. Facial emotional expressions usually reflect someone's emotional state and are part of social communication. Fast early perceptual processing of emotional facial expressions involves the thalamus, amygdala, superior colliculus, and striate cortex [16]. Detailed perception and conceptualization of the emotion signaled by the face engages the striate cortex, fusiform face area, superior temporal gyrus (STG), amygdala, orbitofrontal cortex (OFC), basal ganglia, hypothalamus, insula and brainstem [17,18]. As part of multiple specialized networks, the amygdala is particularly important for the recognition of fear [19], the anterior insula for disgust [20–22], the ventral striatum and the OFC for anger [23], and the amygdala along with the supplementary motor area for the recognition of happiness [24, 25].

The aim of this study was to better specify the early emotion recognition deficits at mild stages of AD and to disentangle their neuroanatomical correlates. We performed facial emotional expression recognition testing in patients diagnosed with AD at the MCI or mild dementia stage, as well as in healthy controls. Only the patient group had 3D structural magnetic resonance (MR) brain imaging. The patients and controls underwent tests to assess their ability to recognize facial expressions of anger, fear, disgust and happiness. Volumes of individual brain regions were obtained by processing the 3D T1-weighted images with MAPER (multi-atlas propagation with enhanced registration) [14, 26]. Correlations between cognitive performance and regional volumes were assessed.

## Material and Methods

This study is part of the PACO (**p**ersonnalité **A**lzheimer **co**mportement—personality, Alzheimer’s disease, and behaviour) programme (Clinical Trial number 01297140), a national collaborative programme evaluating patients diagnosed with AD at the MCI or mild dementia stages. PACO prospectively explores the role of personality and social clue recognition abilities in the occurrence of behavioural disorders later in the disease course.

### Participants

Thirty-nine patients diagnosed with AD at the amnestic MCI stage [11] (n = 15) or the mild dementia stage [27] (n = 24) and 39 healthy control subjects took part in this study. All participants were diagnosed by a board-qualified neurologist with extensive experience in neurodegenerative diseases. Patients were diagnosed either with mild stage dementia according to the Clinical Dementia Rating (CDR) 1 & 2 [28], or with prodromal AD. Patients with prodromal AD, corresponding to amnestic mild cognitive impairment (aMCI) [29] had a clinical dementia rating criteria of 0.5 as set out in the PréAL study and the revised NINCDS-ADRDA diagnostic criteria suggested for research purposes [30]. Patients scored 20/30 or higher on the Mini Mental State Examination (MMSE [31]); control subjects scored 28/30 or higher.

Further inclusion criteria were age (> 50 years), sufficient visual, auditory and French language skills to complete the clinical evaluations, and the availability of a caregiver able to report on patient personality and behaviour. Patients had no progressive or poorly managed psychiatric pathology, were not taking any antipsychotic or psychotropic medication unless chronically and at stable doses, had no evidence for non-AD related disease and were not depressed. All patients enrolled in the PACO program so far who had MR data available were included in the present study.

Inclusion required written consent from both the patient and a caregiver who was closely involved with the patient. In compliance with French law, the study protocol, inclusion criteria, and consent procedure were reviewed and approved by the responsible regional ethics committee (*Comité de Protection des Personnes* South-East III) and the *Agence Nationale de Sécurité du Médicament et des Produits de Santé*. Participation was not remunerated.

Participants’ demographical data are presented in Table 1.

**Table 1:** Demographical and neuropsychological characteristics of the studied sample.

	Controls	Patients	Controls vs patients
**Number**	**39**	**39**	**ns**
**Male / Female**	**13 / 26**	**19 / 20**	**ns**
**Age [min–max]**	**70.2 (7.2) [59–87]**	**79.9 (4.7) [70–88]**	**p<10−7**
**Education level [min–max]**	**3.5 (0.7) [1–4]**	**2.6 (0.9) [1–4]**	**p<10−5**
**MMS (score/30) [min–max]**	**29.3 (0.7) [28–30]**	**23.9 (2.5) [20–29]**	**p<10−12**

Values are presented as mean *(SD)*. The two groups were compared using Student’s t-test (education level) or the Wilcoxon test (age and MMS). Education level: 1: no study degree, 2: “certificat d’études” (5 years of formal education), 3: “brevet” (9 years of formal education), 4: baccalaureate (12 years of formal education) or higher. MMS: Mini-mental state examination. Patients: AD at the MCI stage or at the mild dementia stage.

Min: minimum, max: maximum.

### Behavioural tasks

For the two cognitive tasks described below, photographs of faces and answer options were displayed on a computer screen. The tasks were not timed, but participants were asked to answer as spontaneously as possible. Participants gave their answer verbally and the examiner selected the chosen label on the computer screen. No feedback on responses was provided. Two of the patients did not participate in the gender recognition task. All of the stimuli were developed, validated and used in a previous study by Bediou et al. (2005) [32].

### Facial gender recognition task

This task measures the ability to discriminate the gender from face images in order to rule out deficits of attention, vision, or face structural encoding. As a control task, it ensured that any deficits observed in the facial emotional expression recognition task were specifically due to an impaired ability to recognize emotions.

Photographic images of males and females were morphed with a gender-neutral face to create a gender continuum. The neutral face was obtained by averaging 20 male and 20 female faces. For each male and each female face, a range of six intensity levels of sex features (0%, 20%, 40%, 60%, 80% and 100%) was obtained by computer graphical manipulation [3]. Ninety-six items using 16 different models (eight women) were randomly presented, and participants were asked to determine whether the face was more feminine or masculine.

### Facial emotional expression recognition task

This task evaluates the recognition of four basic emotions: happiness, anger, disgust, and fear [3]. Photographs of facial expressions were morphed with photographs of neutral expressions to create images representing varying degrees (20, 40, 60, 80, or 100%) of the emotional expression. Twenty items for each emotion and 16 neutral faces were presented in random order. Participants were asked to select the label that best described the emotional expression, i.e. “anger”, “fear”, “disgust”, “happiness”, or “neutral”.

We used various morphing degrees as we hypothesized that early-stage disease might only affect recognition of the subtlest facial emotional expressions (20% of intensity for instance), while recognition of more distinct expressions might still be intact.

The unprocessed results spreadsheet of the behavioural tests is provided as a supplement (S1 File).

### Structural MR image processing

#### MR image acquisition

Cranial MR scanning was performed at the time of diagnosis or no later than three months after the cognitive tasks, and only on patients. All sequences (3D T1, axial T2, T2* and FLAIR) were acquired at 1.5 Tesla Philips Achieva scanners at university hospitals in Lyon and Saint-Etienne. A research engineer set up the exact same MR imaging protocol on both sites to ensure that acquisition parameters were identical at both participating hospitals. For volumetric analyses, 1 mm isotropic 3D T1 sequences without contrast injection were performed.

Since our control group did not undergo MR scanning, we used healthy subjects who participated in the Alzheimer’s Disease Neuroimaging Initative (ADNI) as a control group to compare the brain volumes of our patient group. The 3D structural MR images from the ADNI database were segmented using the same procedures as for the patient group. We selected a group of controls with the same age distribution and gender proportions. We only selected controls who scored 28 or more at the MMSE, and whose MR images were acquired on a 1.5 Tesla scanner. From 254 ADNI subjects matching these criteria, we randomly selected 70 for our control group. Two of the controls were removed on an additional exclusion criterion of having one or more brain regions whose volumes were smaller than the mean of the control group by more than three standard deviations.

Data used in the preparation of this article were obtained from the Alzheimer’s Disease Neuroimaging Initiative (ADNI) database (adni.loni.usc.edu). The ADNI was launched in 2003 as a public-private partnership, led by Principal Investigator Michael W. Weiner, MD. The primary goal of ADNI has been to test whether serial MR imaging, positron emission tomography (PET), other biological markers, and clinical and neuropsychological assessment can be combined to measure the progression of MCI and early AD.

#### Image preprocessing

T1-weighted MR images were preprocessed with FSL FAST to correct for intensity variations due to field inhomogeneity, and to obtain masks of the brain and the intracranial space. We first applied pincram, a multi-atlas procedure for brain masking [33], with parameters chosen so as to rapidly obtain a coarse, generous brain label. This label was applied to the original image, resulting in a masked version which was subjected to bias correction with FSL FAST.

Corrected images were processed with pincram to label the intracranial space. Pincram requires atlas data (T1-weighted images with reference labels). A suitable atlas for intracranial masking was obtained by selecting subjects from the IXI cohort (n = 29, http://brain-development.org/) with intracranial masks obtained through reverse brain masking [34]. In a subset of the resulting pincram extractions of the PACO images (n = 18), we observed segmentation errors (partial exclusion of gyral crowns and partial inclusion of meninges). We therefore generated new atlases for pincram using PACO images where the extraction was rated successful on visual review (n = 21). These secondary *PACO-customized atlases* were then applied to all PACO images. No underinclusion problems were subsequently detected on visual review. The resulting brain masks included most of the peripheral cerebrospinal fluid (CSF), even in subjects with substantial brain atrophy. They were thus suitable for estimating the intracranial volume. However, the region anterior to the brainstem was frequently included in the mask, leading to mesiotemporal overestimations. Tighter masks labeling the brain rather than the intracranial space were thus additionally required.

To obtain accurate regional brain labels for the 39 PACO subjects, the images were pincram-extracted using the Hammers atlases [26, 35]. These atlases were generated by manual labeling of 83 brain regions in 30 T1-weighted cranial images of adults. With this extraction, most of the peripheral CSF was excluded from the label and the mask followed the gyral surface with high accuracy. However, for eight subjects, the procedure resulted in substantial over- or underinclusions. In seven of these cases, we reverted to the masks obtained with the *PACO-customized atlases*. In one subject, a suitable image could not be recovered because of substantial differences in the acquisition parameters. This subject was finally excluded.

#### MAPER processing to segment brains into regions of interest

**Fig 1:**
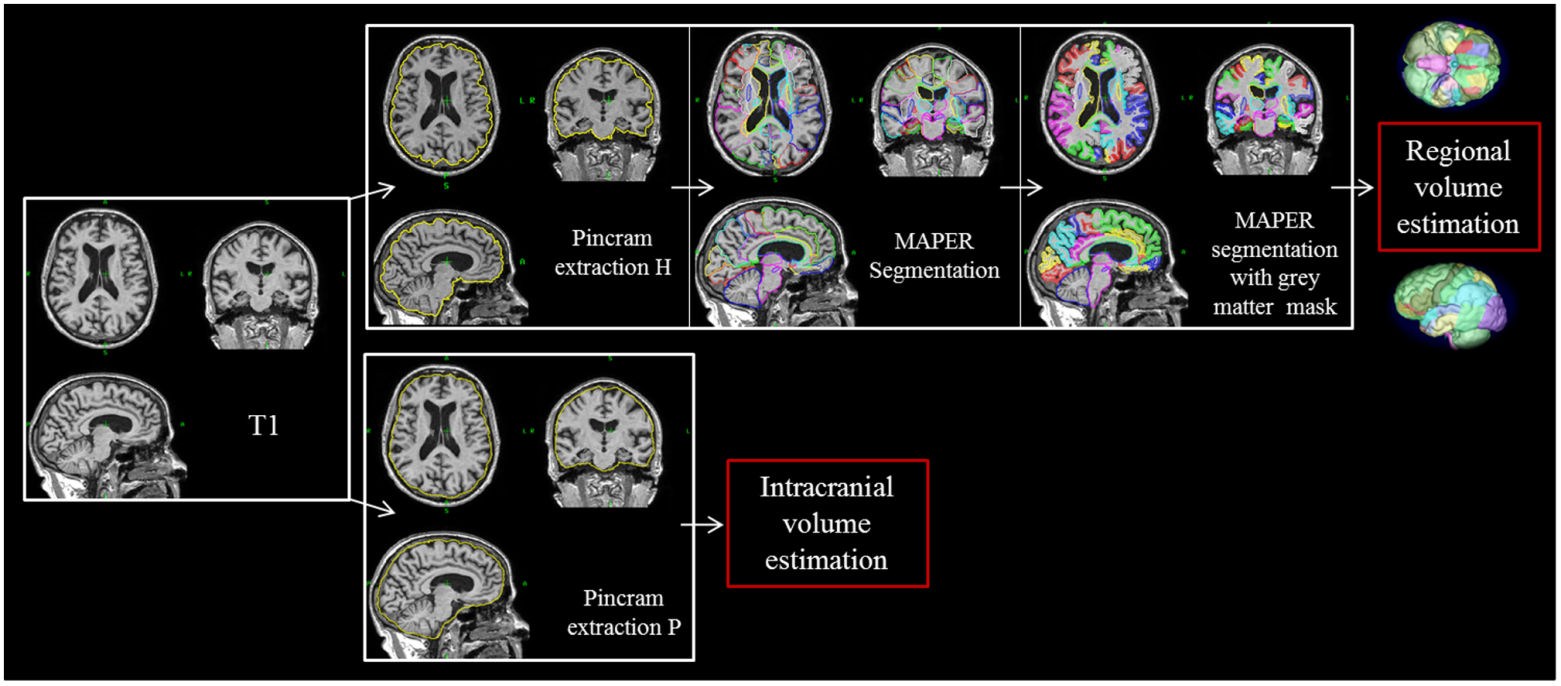
Pincram brain extraction and MAPER segmentation. MRI T1 sequences were processed with pincram, followed by MAPER and grey matter masking to obtain regional grey matter volumes. Extraction P was used to estimate the intracranial volume. *Pincram extraction* ***H***: Brain extraction using the Hammers atlases; *Pincram extraction* ***P***: Brain extraction using the PACO-customized atlases.

MAPER [36] was used to segment the PACO brain images into multiple regions (Fig 1). MAPER has been shown to yield robust results, even for subjects with ventriculomegaly, which occurs frequently in MCI and mild AD, and yields accurate segmentations for images obtained in multicenter settings [14]. MAPER uses input in the form of multi-subject, multi-region input atlases. Here, we used the Hammers atlases (thirty subjects, manually segmented into 83 regions according to strict protocols) [26, 35]. Tissue classification information is incorporated into the image registration process. A grey matter mask based on FSL FAST tissue classification was applied to the 83 regions, except for pure grey matter regions, i.e. the basal ganglia and the amygdala. Regional volumes obtained were normalize through division by the intracranial volume. Selected regions of interest were additionally analyzed volumetrically without this normalization.

Every segmented brain image was visually checked, especially in the regions of interest. In addition, the regions with the smallest and largest volumes were visually checked on the corresponding image to ensure that their segmentation was accurate.

Preprocessed MR images as subjected to analysis, along with segmentation results, are publicly available [37].

### Statistical analysis

The variables of interest showed no difference in variance (tested using Fisher variance test) and were normally distributed (tested using the Shapiro test). Thus, they were suitable for parametric analyses.

Demographical and cognition variables were analyzed using two-tailed Student's *t*-tests or Wilcoxon tests when comparing the patient group (MCI and mild AD) with the control group. The groups were well matched for sex, but differed significantly in age (patients were older, p<10–5) and education level (patients had lower education level, p<10–5). To take this difference into account, analysis of covariance (ANCOVA) was applied with age, sex, and education level as a covariate. Correlations between performance on the emotion recognition task and volumes of brain regions were examined using Pearson analysis. We studied correlations based on our regions of interest, and corrected for multiple comparisons using Benjamini & Hochberg’s method [38]. Nine bilateral regions were investigated for anger recognition (putamen, posterior STG, fusiform gyrus, anterior cingulate gyrus (ACG), precentral gyrus, anterior orbital gyrus, inferior frontal gyrus (IFG), gyrus parahippocampalis, lingual gyrus), seven bilateral regions for disgust (insula, caudate nucleus, putamen, pallidum, IFG, posterior STG, ACG), ten bilateral regions for fear recognition (amygdala, hippocampus, posterior STG, fusiform gyrus, ACG, thalamus, anterior orbital gyrus, IFG, medial orbital gyrus, lateral orbital gyrus) and seven bilateral regions for happiness recognition (amygdala, fusiform gyrus, insula, ACG, anterior orbital gyrus, lingual gyrus, medial orbital gyrus). All statistical analyses were performed using R (http://www.r-project.org). The threshold for statistical significance was set at p<0.05.

Previously, Perrin et al. [39] had manually segmented the amygdala of ten of our patients using a different protocol to that used for the Hammers atlases. Their measurements of amygdala volumes were systematically larger but strongly consistent with the MAPER measurements (right amygdala: ICC 0.84; left: ICC = 0.86).

## Results

### Behavioural tasks

#### Gender recognition performance

**Fig 2:**
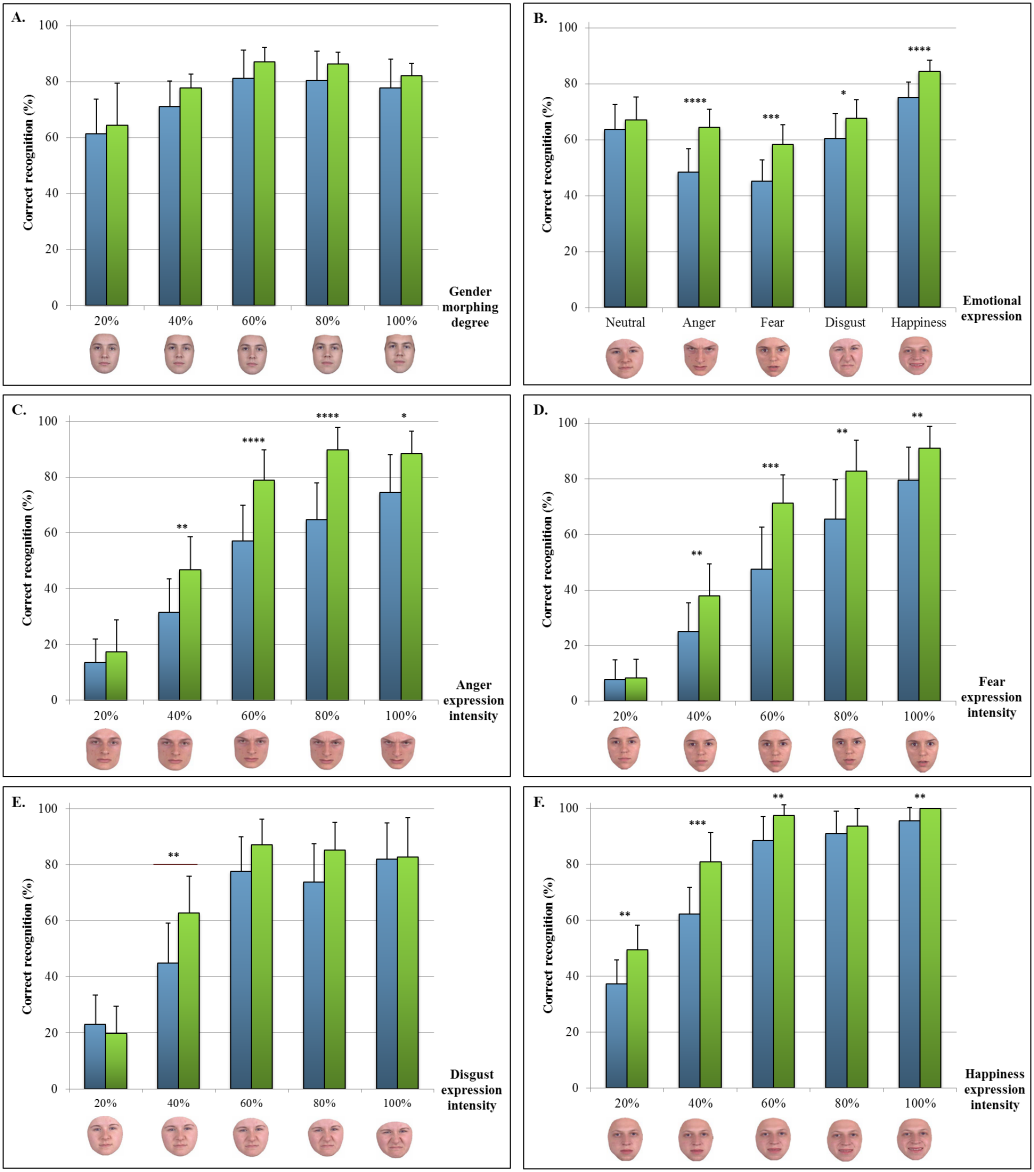
Mean performance on cognitive tasks. A: Gender recognition; B: Emotion recognition; C: Anger recognition; D: Fear recognition; E: Disgust recognition; F: Happiness recognition. Examples of morphed faces depicting various intensities of the emotional expression are shown at the bottom of each graph. Blue: patients (AD at the MCI stage or at the mild dementia stage); Green: controls. *p <.05; **p<10–2; ***p<10–3; ****p<10–4.

As expected, no significant difference was found between the patient and the control groups for any of the conditions on the gender recognition task (patients: 74% in average, controls: 79% in average) (Fig 2A).

#### Emotion recognition performance

On average, patients performed below controls on emotion recognition (57% vs 69%, p<10^−10^). The two groups showed similar performance on the recognition of neutral expressions (p>0.05). When averaging the five degrees of morphing for each emotion, we found the patient group to be significantly impaired in the recognition of anger (mean performance: 48% vs. 64%, p<10^−4^), fear (mean performance: 45% vs. 58%, p<10^−4^), disgust (mean performance: 60% vs 68%, p = 0.049), and happiness (mean performance: 75% vs 84%, p<10^−4^) (Fig 2B).

#### Detailed analysis of individual emotions by degree of morphing intensity

Analysis of individual emotions revealed a variable pattern (Fig 2C to 2F). In comparison to the control group, the patient group was impaired in anger recognition with morphing intensities of 40–100% (from p<0.02 to p<10^−4^), fear recognition with morphing intensities of 40–100% (from p<10^−2^ to 10^−4^), disgust recognition with morphing intensity of 40% (p<10^−2^), and happiness recognition with morphing intensities of 20–60% (from p<10^−2^ to p<10^−4^) and 100% (p<10^−2^).

#### Covariance with demographical data

Analysis including all participants revealed that global emotion recognition performance deteriorated with age (r = −0.42). Age was negatively correlated with recognition of anger (r = −0.38), fear (r = −0.30), and happiness (r = −0.30).

#### Structural imaging findings

Regional segmentation succeeded on every region, except for the cerebellum, which appeared to be slightly underestimated in some subjects. Using MAPER, we segmented 76 regional volumes corresponding to the 83 regions without the cerebellum and ventricles, and considered our regions of interest defined prior to the segmentation. Shapiro tests indicated that the volumes of the regions of interest were normally distributed.

### Neuroanatomical correlates

#### Correlations with emotion recognition performance

In the following analysis, the impaired recognition (IR) condition corresponds to the patients’ average emotion recognition performance at morphing degrees where they performed significantly below controls. For instance, anger IR corresponds to the patients’ average performance on anger recognition with morphing degrees of 40, 60, 80, and 100%.

**Fig 3:**
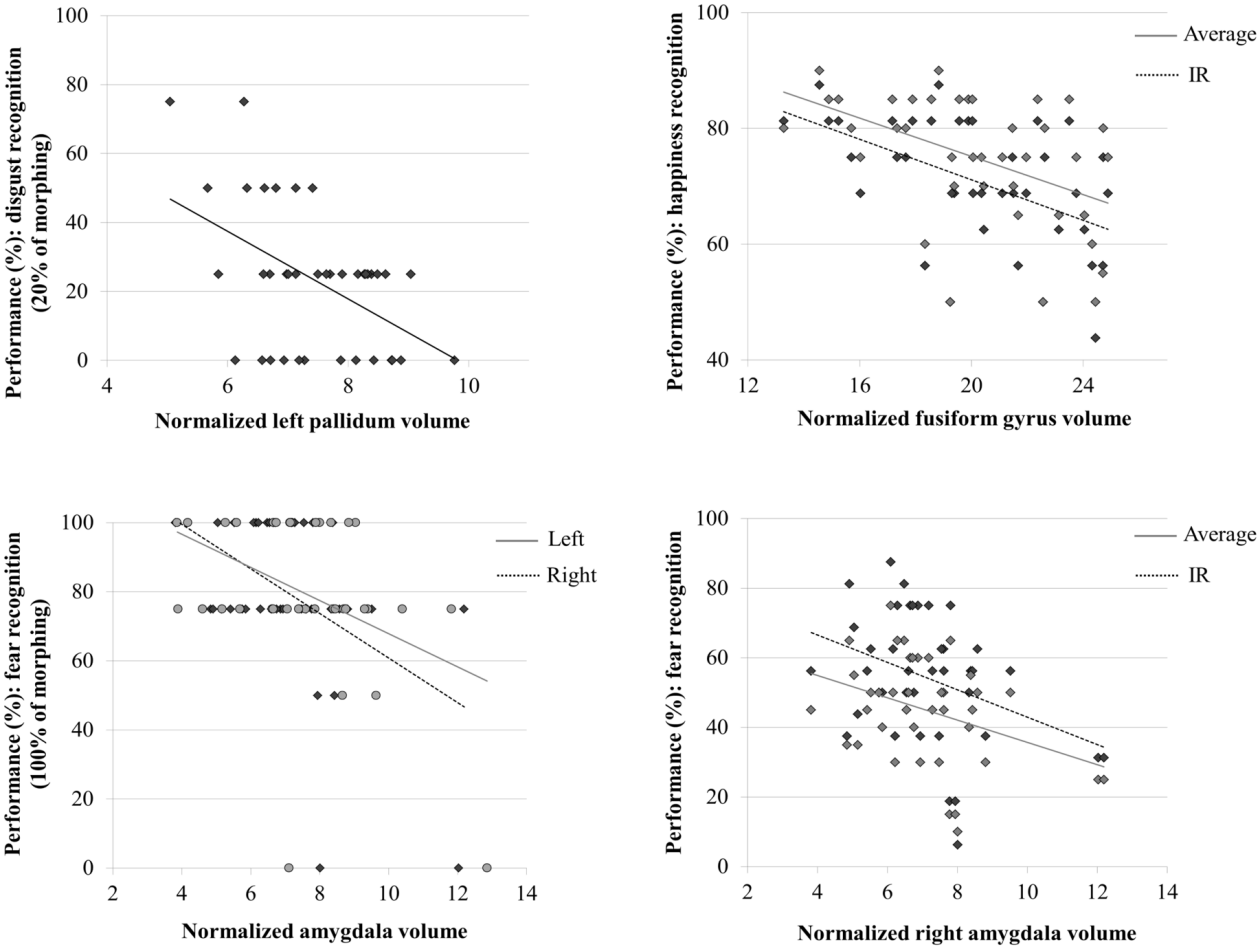
Neuroanatomical correlates of emotion recognition in patients with AD at the MCI stage or at the mild dementia stage. The graphics show the significant correlations between regional volumes normalized by the intracranial volume and performance for disgust recognition (A), happiness recognition (B), and fear recognition (C-D). IR: impaired recognition condition. Measurements were obtained using Pearson’s correlation tests.

We investigated correlations between brain regional volume and performance and corrected for multiple comparisons as described in the Methods section (Table 2). Fear performance was negatively correlated with the right and left amygdala volumes for varying degrees of morphing (Fig 3C and 3D). Disgust (with 20% of morphing) was negatively correlated with the volume of the left pallidum (Fig 3A). Happiness recognition was negatively correlated with the volume of the left fusiform gyrus (Fig 3B). No significant correlations were revealed with anger recognition when correcting for multiple comparisons. However, without this level of correction, negative correlations between anger recognition and the right lingual gyrus volume were seen (average of morphing degrees and IR: both r = −0.42, p = 0.008).

**Table 2:** Correlations between emotion recognition performance and regional volumes in the patient group.

Brain region	Facial expression	Condition	Side	r	p-value	
					*uncorrected*	*corrected*
**Amygdala**	Fear	100% of morphing	L	-0.40	p<10^−3^	p = 0.009
			R	-0.47	p<10^−3^	p = 0.007
		Average	R	-0.36	p = 0.002	p = 0.047
		IR	R	-0.36	p = 0.002	p = 0.046
**Fusiform G.**	Happiness	Average	L	-0.47	p = 0.003	p = 0.037
		IR	L	-0.51	p = 0.001	p = 0.015
**Pallidum**	Disgust	20% of morphing	L	-0.49	p = 0.002	p = 0.022

An overall analysis of the 76 regions without correction for multiple comparisons revealed that most of the volumes of brain regions were negatively correlated with performance on the emotion recognition tasks (cf. Supporting Information). The negative correlations between fear recognition (100% of morphing) and amygdala volumes remained significant after Benjamini-Hochberg correction for 76 comparisons.

The correlations between fear IR performance and the amygdala volumes were compared to those derived from the manually determined amygdala volumes [38] after normalization of the latter by intracranial volume (n = 10). These manually determined volumes were also negatively correlated with the recognition of fear 100% (right: r = −0.72, p = 0.018; left: r = −0.78, p<10^−2^).

To exclude the possibility that the correlations were an artifact of normalization by intracranial volume, we repeated the analysis with non-normalized volumes and obtained very similar results (data not shown).

#### Comparison between MCI and AD

We repeated the correlation analysis of the previous subsection after having grouped the patients by diagnosis (Table 3). Even when analyzed separately, the two groups showed the correlation (albeit with lower significance, as was to be expected due to the sample size reduction).

**Table 3:** Correlations between emotion recognition performance and regional volumes, separated by diagnosis (AD/MCI).

Brain region	Facial expression	Condition	Side	Patients with MCI		Patients with mild AD	
				r	p	r	p
Amygdala	Fear	100% of morphing	L	-0.73	0.002	-0.09	0.68
			R	-0.79	0.0005	-0.22	0.29
		Average	R	-0.42	0.12	-0.32	0.12
		IR	R	-0.46	0.087	-0.31	0.13
Fusiform G.	Happiness	Average	L	-0.66	0.007	-0.29	0.17
		IR	L	-0.72	0.002	-0.32	0.13
Pallidum	Disgust	20% of morphing	L	-0.55	0.034	-0.50	0.01

#### Correlations between regions of interest

**Fig 4:**
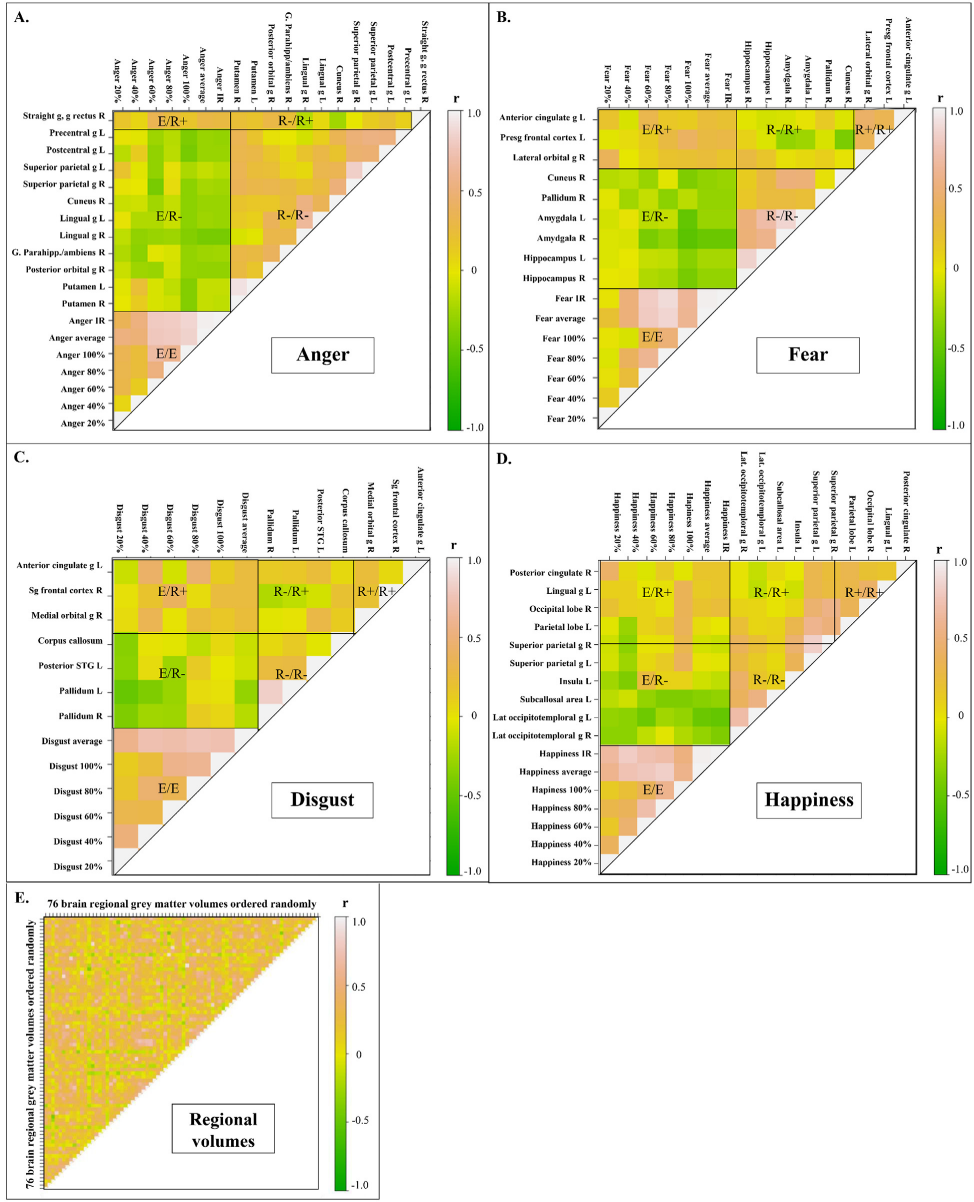
Correlations between neuroanatomical structure volumes and emotion recognition performance. The plots show the correlation coefficients (r) between the different conditions. Only the regions which were significantly correlated (uncorrected p-values) with the recognition of an emotion are presented. E = Emotion recognition performance; R+ = Regional volume positively correlated with the recognition of emotions; R- = Regional volume negatively correlated with the recognition of emotions; e.g. E/R+ = correlation between emotion recognition performance and regional volumes positively correlated with the recognition of emotions. R = right, L = left, IR: impaired recognition.

These exploratory correlations were performed considering the uncorrected comparison results (Supporting Information). The regional volumes which were positively correlated with the recognition of an emotion were also positively correlated among themselves (average r = 0.28, p<10^−6^). Similarly, the regional volumes that were negatively correlated with the recognition of an emotion were positively correlated among themselves (average r = 0.28, p<10^−6^). Regional volumes positively correlated with the recognition of an emotion were only weakly correlated with the regions negatively correlated with the recognition of the same emotion (average: r = 0.12, p<10^−6^).

(Fig 4A to 4D) shows the correlation coefficients between emotion recognition performance, regional volumes positively correlated with the recognition of emotions, and regional volumes negatively correlated with the recognition of emotions. When plotting the 76 regions of interest in random order, no clear pattern of correlation is evident (Fig 4E).

#### Region volume comparison with healthy controls

To exclude the possibility that our patient group was unusual with regard to the region volumes we determined, we compared measurements on selected regions (right and left amygdala, left fusiform, left pallidum, and hippocampus left and right mean) with those obtained on the control group selected from the ADNI database. The volumes were significantly smaller in patients than in controls, except for the fusiform and the pallidum. The fusiform was smaller, but not significantly so. The pallidum was larger by 13%; however pallidum measurements tend to be inaccurate with MAPER [14]. All other structures were approximately 25% smaller. These findings are consistent with moderate atrophy as expected in a patient group.

## Discussion

This study revealed specific impairments in facial emotion expression recognition in patients with AD at the MCI or mild dementia stages, whereas recognition of other facial features like gender remains unimpaired. This is consistent with a selective impairment of recognition of facial features relying on temporal and frontal neural networks. Features that are invariant to emotion, like those identifying gender, are still recognized, as this ability depends on posterior occipital areas that are fully or largely preserved during early stages of AD [12]. We found correlations between emotion recognition performance and the volumes of the expected brain structures. However, a new finding was that emotion recognition performance was negatively correlated with most of the grey matter volumes of regions of interest. In particular, fear recognition performance was negatively correlated with the amygdala volumes, whether these were measured automatically or manually. These unexpected results mean that larger regional volumes are associated with lower performance. We had initially hypothesized that the most atrophied regions would be associated with the most impaired cognitive functions.

### Emotion impairment in mild stages of AD

#### Recognition of emotion is impaired in mild stages of AD

We found that emotion recognition was impaired during mild stages of AD. Previous reports on early AD identified distinct deficits in episodic memory and milder deficits of executive, language and visual functions [4]. Some epidemiological studies have highlighted unexpected signs at very early stages of AD such as impaired semantic abilities [4], and this may help to understand and characterize clinical presentation at the predementia stage. Demonstrating early changes in social cognition in AD may be essential for understanding the social disinvestment observed in the first stages of the disease.

Unlike Spoletini et al. [8], who found that amnestic MCI patients were only impaired in the recognition of low-intensity fearful faces, we found that patients were impaired in the recognition of each of several different emotions (anger, fear, happiness, and, to a lesser degree, disgust). Such results were also shown by Bediou et al. [3].

#### Disgust recognition is selectively preserved

Disgust recognition appeared to be selectively preserved, except at the morphing degree of 40%. This is congruent with the study by Henry et al. [5] and may be explained by the relative preservation of the basal ganglia in AD [40], despite significant atrophy of the insula, the two structures previously found to play a role in disgust recognition [41]. Bediou et al. had found that disgust recognition was preserved in MCI but not in mild AD, which is partially consistent with our results [3].

#### The impairment is specific to emotion recognition

Patients were neither impaired on gender recognition, nor on neutral expression recognition. Thus, their ability to recognize facial features invariant to emotion was preserved, as well as their visual and attention skills. Our results indicate that in MCI and mild AD, emotion recognition is impaired specifically, rather than as a consequence of global cognitive dysfunction.

### Neuroanatomical correlates of emotion recognition

The correlations between emotion recognition performance and regional volumes revealed networks encompassing the regions for which involvement was predicted, even though the direction of the correlation was unexpected. Our findings showing negative correlations between the amygdala volumes and the recognition of fear 100% were confirmed by the manual segmentation of the amygdala [39] and persisted even after correction for multiple comparisons. To our knowledge, previous studies have not investigated the neuroanatomical correlates of emotion recognition in the mild stages of AD in such detail.

The total emotion recognition score was negatively correlated with the amygdala volume (bilaterally) and the left hippocampal volume, and was positively correlated with the left anterior cingulate gyrus and the right medial orbital gyrus volumes. These four regions are activated in subjects looking at emotionally expressive faces [17]. Regional involvement was entirely consistent with our hypotheses: anger recognition correlated with the OFC volume as well as the ventral striatum volume, fear recognition with the amygdala volume, and happiness recognition with the fusiform gyrus volume.

We did not find the expected correlation between the insula volume and disgust recognition. Disgust recognition is processed in the ventral anterior subregion of the insula. The 83-region Hammers atlases currently label the insula as a whole, which may explain the absence of a correlation in our study.

### Hypotheses to explain negative correlations

Contrary to our expectations, we only found negative correlations between emotion recognition performance and grey matter volumes of a priori specified regions of interest. Some hypotheses are presented below to explain why patients with larger volumes may show reduced performance.

The traditional interpretation of atrophy of these regions leading to impaired cognitive abilities is regularly found correct in later stages of Alzheimer’s disease [6], when e.g. hippocampus volume is positively correlated with memory performance [42, 43].

#### Amyloid plaque bulk hypothesis

A study by Bussière et al. [44] estimated the volume occupied by amyloid deposits in the hippocampus, the entorhinal cortex, and area 9 of the frontal cortex. On average, the β-amyloid volume represented 7.3% of the regional volume, with values ranging from 0% to 34.8% [44]. Moreover, patients who had received anti-β amyloid treatment with AN1792 to eradicate amyloid plaques showed decreased regional volumes. Evidently, amyloid plaques occupy space and increase regional volumes. At early stages of the disease, the amount of amyloid deposits is thus correlated with some cognitive impairment, in particular memory, and hippocampal atrophy [45]. In order to validate this hypothesis, one might use positron emission tomography with an amyloid tracer to estimate regional β-amyloid burden.

#### Neuroinflammation hypothesis

In mild AD, amyloid plaques are associated with an inflammatory response involving an increased presence of activated complement proteins, cytokines, and activated microglia and astrocytes. Neuroinflammation occurs mostly in the frontal neocortex and limbic system, and precedes cerebral atrophy. The volume occupied by microglia and macrophages increases with the severity of the dementia [46].

It is therefore possible that the regions with the largest volumes are those with the highest degree of neuroinflammation. This hypothesis could be tested by assessing the severity of neuroinflammation by positron emission tomography with markers for activated microglia, e.g. [11C]PK11195.

As neuroinflammation may be correlated with β-amyloid burden [47], the neuroinflammation and amyloid hypotheses both predict increased regional volume to be correlated with cognitive impairment [48].

#### Hypercompensation hypothesis

In MCI, functional compensatory mechanisms [49] involving up-regulation of neurotransmitter receptors [50] or enzymes [51] can occur. For instance, increased hippocampal activation was reported in the MCI stage of AD compared to controls [52]. Structural compensation with neuronal hypertrophy in neurodegenerative diseases has also been suggested [53]. The most functionally impaired regions may be structurally compensating the most.

Our findings also point out the fact that, with MCI patients, structural MRI might not inform properly about the impairments encountered by the patients. Indeed, at the first stages of AD, neuronal death might be underestimated on MR scans since processes like inflammation or amyloid deposits might contribute to relative preservation of some brain region volumes. A complementary method such as PET with selective ligands may help to disentangle these pathological mechanisms.

### Emotion recognition network

Structural co-variance is increasingly recognized as relevant for studying brain function in health and disease [54]. We found evidence for structural co-variance in emotion recognition networks in our study. Fig 4 illustrates that regions for which the correlation with emotion recognition has the same direction are positively correlated amongst themselves, whereas no clear correlations were seen when considering all regions of interest in random order. This may indicate that regional involvement in each kind of emotion recognition follows distinct patterns, which supports the locationist view that postulates unique regional networks for each emotion [17].

#### Limitations and perspectives

The patient group consisted of a mixed population of MCI and AD at mild stage, even if the MCI patients included were typical of the form converting into AD. Moreover, controls did not undergo MRI, which prevented volume comparisons between the two groups. MAPER has previously been shown to robustly identify signature structural differences between healthy controls and MCI and AD patients [14].

In future studies, structural connectivity using DTI between the regions of interest could be explored. The functional overactivation hypothesis could be explored with functional MRI, the β-amyloid burden with amyloid PET, or the neuroinflammation hypothesis with activated microglia PET.

## Supporting Information

S1 File Results of neuropsychological testing (unprocessed). Column A is an identifying number *n* that connects the patients (upper set of 39 data rows) with their imaging data files [37] via the filename (pattern c*n*.nii.gz).
(XLS)

S1 Table Correlations between emotion recognition performance and regional volumes in the patient group. R: right, L: left. IR: impaired recognition. r: Pearson correlation coefficient. Green = negative correlations, red = positive correlations. *p<0.05, **p<10–2, ***p<10–3. Literature: Region hypothesized to be involved in task *a priori*. These correlations were not corrected for multiple comparisons; compare with Table 2 in the main article.
(DOCX)

## References

[1] Hargrave R, Maddock RJ, Stone V. Impaired recognition of facial expressions of emotion in Alzheimer’s disease. J Neuropsychiatry Clin Neurosci. 2002;14: 64–71. 11884657 10.1176/jnp.14.1.64

[2] Kohler CG, Anselmo-Gallagher G, Bilker W, Karlawish J, Gur RE, Clark CM. Emotion-discrimination deficits in mild Alzheimer disease. Am J Geriatr Psychiatry Off J Am Assoc Geriatr Psychiatry. 2005;13: 926–933. 10.1176/appi.ajgp.13.11.926 16286435

[3] Bediou B, Ryff I, Mercier B, Milliery M, Hénaff M-A, D’Amato T, et al Impaired social cognition in mild Alzheimer disease. J Geriatr Psychiatry Neurol. 2009;22: 130–140. 10.1177/0891988709332939 19321881

[4] Amieva H, Le Goff M, Millet X, Orgogozo JM, Pérès K, Barberger-Gateau P, et al Prodromal Alzheimer’s disease: successive emergence of the clinical symptoms. Ann Neurol. 2008;64: 492–498. 10.1002/ana.21509 19067364

[5] Henry JD, Ruffman T, McDonald S, O’Leary M-AP, Phillips LH, Brodaty H, et al Recognition of disgust is selectively preserved in Alzheimer’s disease. Neuropsychologia. 2008;46: 1363–1370. 10.1016/j.neuropsychologia.2007.12.012 18241898

[6] Kumfor F, Sapey-Triomphe L-A, Leyton CE, Burrell JR, Hodges JR, Piguet O. Degradation of emotion processing ability in corticobasal syndrome and Alzheimer’s disease. Brain J Neurol. 2014; 10.1093/brain/awu246 25227744

[7] Teng E, Lu PH, Cummings JL. Deficits in facial emotion processing in mild cognitive impairment. Dement Geriatr Cogn Disord. 2007;23: 271–279. 10.1159/000100829 17351319

[8] Spoletini I, Marra C, Di Iulio F, Gianni W, Sancesario G, Giubilei F, et al Facial emotion recognition deficit in amnestic mild cognitive impairment and Alzheimer disease. Am J Geriatr Psychiatry Off J Am Assoc Geriatr Psychiatry. 2008;16: 389–398. 10.1097/JGP.0b013e318165dbce 18403572

[9] Weiss EM, Kohler CG, Vonbank J, Stadelmann E, Kemmler G, Hinterhuber H, et al Impairment in emotion recognition abilities in patients with mild cognitive impairment, early and moderate Alzheimer disease compared with healthy comparison subjects. Am J Geriatr Psychiatry Off J Am Assoc Geriatr Psychiatry. 2008;16: 974–980. 10.1097/JGP.0b013e318186bd53 19038896

[10] McCade D, Savage G, Naismith SL. Review of emotion recognition in mild cognitive impairment. Dement Geriatr Cogn Disord. 2011;32: 257–266. 10.1159/000335009 22222774

[11] Albert MS. Changes in cognition. Neurobiol Aging. 2011;32 Suppl 1: S58–63. 10.1016/j.neurobiolaging.2011.09.010 22078174 PMC3929949

[12] Chetelat G, Baron J-C. Early diagnosis of Alzheimer’s disease: contribution of structural neuroimaging. NeuroImage. 2003;18: 525–541. 12595205 10.1016/s1053-8119(02)00026-5

[13] Whitwell JL, Petersen RC, Negash S, Weigand SD, Kantarci K, Ivnik RJ, et al Patterns of atrophy differ among specific subtypes of mild cognitive impairment. Arch Neurol. 2007;64: 1130–1138. 10.1001/archneur.64.8.1130 17698703 PMC2735186

[14] Heckemann RA, Keihaninejad S, Aljabar P, Gray KR, Nielsen C, Rueckert D, et al Automatic morphometry in Alzheimer’s disease and mild cognitive impairment. NeuroImage. 2011;56: 2024–2037. 10.1016/j.neuroimage.2011.03.014 21397703 PMC3153069

[15] Lindquist KA, Wager TD, Kober H, Bliss-Moreau E, Barrett LF. The brain basis of emotion: a meta-analytic review. Behav Brain Sci. 2012;35: 121–143. 10.1017/S0140525X11000446 22617651 PMC4329228

[16] Adolphs R. Recognizing emotion from facial expressions: psychological and neurological mechanisms. Behav Cogn Neurosci Rev. 2002;1: 21–62. 17715585 10.1177/1534582302001001003

[17] Adolphs R. Neural systems for recognizing emotion. Curr Opin Neurobiol. 2002;12: 169–177. 12015233 10.1016/s0959-4388(02)00301-x

[18] Vuilleumier P, Pourtois G. Distributed and interactive brain mechanisms during emotion face perception: evidence from functional neuroimaging. Neuropsychologia. 2007;45: 174–194. 10.1016/j.neuropsychologia.2006.06.003 16854439

[19] LeDoux J. The emotional brain, fear, and the amygdala. Cell Mol Neurobiol. 2003;23: 727–738. 14514027 10.1023/A:1025048802629 PMC11530156

[20] Krolak-Salmon P, Hénaff M-A, Isnard J, Tallon-Baudry C, Guénot M, Vighetto A, et al An attention modulated response to disgust in human ventral anterior insula. Ann Neurol. 2003;53: 446–453. 10.1002/ana.10502 12666112

[21] Wicker B, Keysers C, Plailly J, Royet J-P, Gallese V, Rizzolatti G. Both of Us Disgusted in My Insula: The Common Neural Basis of Seeing and Feeling Disgust. Neuron. 2003;40: 655–664. 10.1016/S0896-6273(03)00679-2 14642287

[22] Sprengelmeyer R, Young AW, Calder AJ, Karnat A, Lange H, Hömberg V, et al Loss of disgust. Perception of faces and emotions in Huntington’s disease. Brain J Neurol. 1996;119 (Pt 5): 1647–1665. 10.1093/brain/119.5.1647 8931587

[23] Blair RJ, Morris JS, Frith CD, Perrett DI, Dolan RJ. Dissociable neural responses to facial expressions of sadness and anger. Brain J Neurol. 1999;122 (Pt 5): 883–893. 10.1093/brain/122.5.883 10355673

[24] Phan KL, Wager T, Taylor SF, Liberzon I. Functional neuroanatomy of emotion: a meta-analysis of emotion activation studies in PET and fMRI. NeuroImage. 2002;16: 331–348. 10.1006/nimg.2002.1087 12030820

[25] Krolak-Salmon P, Hénaff MA, Bertrand O, Vighetto A, Mauguière F. Les visages et leurs émotions: Partie II : La reconnaissance des expressions faciales. Rev Neurol (Paris). 2006;162: 1047–1058. 10.1016/S0035-3787(06)75117-7 17086141

[26] Gousias IS, Rueckert D, Heckemann RA, Dyet LE, Boardman JP, Edwards AD, et al Automatic segmentation of brain MRIs of 2-year-olds into 83 regions of interest. NeuroImage. 2008;40: 672–684. 10.1016/j.neuroimage.2007.11.034 18234511

[27] McKhann GM, Knopman DS, Chertkow H, Hyman BT, Jack CR Jr, Kawas CH, et al The diagnosis of dementia due to Alzheimer’s disease: recommendations from the National Institute on Aging-Alzheimer’s Association workgroups on diagnostic guidelines for Alzheimer’s disease. Alzheimers Dement J Alzheimers Assoc. 2011;7: 263–269. 10.1016/j.jalz.2011.03.005 PMC3312024 21514250

[28] Hughes CP, Berg L, Danziger WL, Coben LA, Martin RL. A new clinical scale for the staging of dementia. Br J Psychiatry J Ment Sci. 1982;140: 566–572. 10.1192/bjp.140.6.566 7104545

[29] Petersen RC, Doody R, Kurz A, Mohs RC, Morris JC, Rabins PV, et al Current concepts in mild cognitive impairment. Arch Neurol. 2001;58: 1985–1992. 11735772 10.1001/archneur.58.12.1985

[30] Dubois B, Feldman HH, Jacova C, Dekosky ST, Barberger-Gateau P, Cummings J, et al Research criteria for the diagnosis of Alzheimer’s disease: revising the NINCDS-ADRDA criteria. Lancet Neurol. 2007;6: 734–746. 10.1016/S1474-4422(07)70178-3 17616482

[31] Folstein MF, Folstein SE, McHugh PR. “Mini-mental state”. A practical method for grading the cognitive state of patients for the clinician. J Psychiatr Res. 1975;12: 189–198. 1202204 10.1016/0022-3956(75)90026-6

[32] Bediou Benoit K-S P. Facial expression and sex recognition in schizophrenia and depression. Can J Psychiatry Rev Can Psychiatr. 2005;50: 525–33. 10.1177/070674370505000905 16262107

[33] Heckemann RA, Ledig C, Gray KR, Aljabar P, Rueckert D, Hajnal JV, et al Brain Extraction Using Label Propagation and Group Agreement: Pincram. PloS One. 2015;10: e0129211 10.1371/journal.pone.0129211 26161961 PMC4498771

[34] Keihaninejad S, Heckemann RA, Fagiolo G, Symms MR, Hajnal JV, Hammers A, et al A robust method to estimate the intracranial volume across MRI field strengths (1.5T and 3T). NeuroImage. 2010;50: 1427–1437. 10.1016/j.neuroimage.2010.01.064 20114082 PMC2883144

[35] Hammers A, Allom R, Koepp MJ, Free SL, Myers R, Lemieux L, et al Three-dimensional maximum probability atlas of the human brain, with particular reference to the temporal lobe. Hum Brain Mapp. 2003;19: 224–247. 10.1002/hbm.10123 12874777 PMC6871794

[36] Heckemann RA, Keihaninejad S, Aljabar P, Rueckert D, Hajnal JV, Hammers A. Improving intersubject image registration using tissue-class information benefits robustness and accuracy of multi-atlas based anatomical segmentation. NeuroImage. 2010;51: 221–227. 10.1016/j.neuroimage.2010.01.072 20114079

[37] Sapey-Triomphe LA, Heckemann RA, Boublay N, Dorey JM, Hénaff MA, Rouch I, Padovan C, Hammers A, Krolak-Salmon P (2015): PACO baseline MR brain images and segmentations. figshare. Available: 10.6084/m9.figshare.1534657 PMC4684414 26673928

[38] Benjamini Y, Hochberg Y. Controlling the False Discovery Rate: A Practical and Powerful Approach to Multiple Testing. J R Stat Soc Ser B Methodol. 1995;57: 289–300.

[39] Perrin M, Henaff M-A, Padovan C, Faillenot I, Merville A, Krolak-Salmon P. Influence of emotional content and context on memory in mild Alzheimer’s disease. J Alzheimers Dis JAD. 2012;29: 817–826. 10.3233/JAD-2012-111490 22349683

[40] Braak H, Braak E. Neuropathological stageing of Alzheimer-related changes. Acta Neuropathol (Berl). 1991;82: 239–259. 1759558 10.1007/BF00308809

[41] Calder AJ, Keane J, Manes F, Antoun N, Young AW. Impaired recognition and experience of disgust following brain injury. Nat Neurosci. 2000;3: 1077–1078. 10.1038/80586 11036262

[42] Petersen RC, Jack CR Jr, Xu YC, Waring SC, O’Brien PC, Smith GE, et al Memory and MRI-based hippocampal volumes in aging and AD. Neurology. 2000;54: 581–587. 10680786 10.1212/wnl.54.3.581

[43] Laakso MP, Soininen H, Partanen K, Helkala EL, Hartikainen P, Vainio P, et al Volumes of hippocampus, amygdala and frontal lobes in the MRI-based diagnosis of early Alzheimer’s disease: correlation with memory functions. J Neural Transm Park Dis Dement Sect. 1995;9: 73–86. 7605591 10.1007/BF02252964

[44] Bussière T, Friend PD, Sadeghi N, Wicinski B, Lin GI, Bouras C, et al Stereologic assessment of the total cortical volume occupied by amyloid deposits and its relationship with cognitive status in aging and Alzheimer’s disease. Neuroscience. 2002;112: 75–91. 12044473 10.1016/s0306-4522(02)00056-8

[45] Mormino EC, Kluth JT, Madison CM, Rabinovici GD, Baker SL, Miller BL, et al Episodic memory loss is related to hippocampal-mediated beta-amyloid deposition in elderly subjects. Brain J Neurol. 2009;132: 1310–1323. 10.1093/brain/awn320 PMC2677792 19042931

[46] Akiyama H, Barger S, Barnum S, Bradt B, Bauer J, Cole GM, et al Inflammation and Alzheimer’s disease. Neurobiol Aging. 2000;21: 383–421. 10858586 10.1016/s0197-4580(00)00124-x PMC3887148

[47] Edison P, Archer HA, Gerhard A, Hinz R, Pavese N, Turkheimer FE, et al Microglia, amyloid, and cognition in Alzheimer’s disease: An [11C](R)PK11195-PET and [11C]PIB-PET study. Neurobiol Dis. 2008;32: 412–419. 10.1016/j.nbd.2008.08.001 18786637

[48] Willis M, Leitner I, Jellinger KA, Marksteiner J. Chromogranin peptides in brain diseases. J Neural Transm Vienna Austria 1996. 2011;118: 727–735. 10.1007/s00702-011-0648-z 21533607

[49] Putcha D, Brickhouse M, O’Keefe K, Sullivan C, Rentz D, Marshall G, et al Hippocampal hyperactivation associated with cortical thinning in Alzheimer’s disease signature regions in non-demented elderly adults. J Neurosci Off J Soc Neurosci. 2011;31: 17680–17688. 10.1523/JNEUROSCI.4740-11.2011 PMC3289551 22131428

[50] Truchot L, Costes SN, Zimmer L, Laurent B, Le Bars D, Thomas-Antérion C, et al Up-regulation of hippocampal serotonin metabolism in mild cognitive impairment. Neurology. 2007;69: 1012–1017. 10.1212/01.wnl.0000271377.52421.4a 17785670

[51] DeKosky ST, Ikonomovic MD, Styren SD, Beckett L, Wisniewski S, Bennett DA, et al Upregulation of choline acetyltransferase activity in hippocampus and frontal cortex of elderly subjects with mild cognitive impairment. Ann Neurol. 2002;51: 145–155. 11835370 10.1002/ana.10069

[52] Dickerson BC, Salat DH, Greve DN, Chua EF, Rand-Giovannetti E, Rentz DM, et al Increased hippocampal activation in mild cognitive impairment compared to normal aging and AD. Neurology. 2005;65: 404–411. 10.1212/01.wnl.0000171450.97464.49 16087905 PMC4335677

[53] Adalbert R, Coleman MP. Axon pathology in age-related neurodegenerative disorders. Neuropathol Appl Neurobiol. 2012; 10.1111/j.1365-2990.2012.01308.x 23046254

[54] Alexander-Bloch A, Giedd JN, Bullmore E. Imaging structural co-variance between human brain regions. Nat Rev Neurosci. 2013;14: 322–336. 10.1038/nrn3465 23531697 PMC4043276

