# Supplementary material for: Neuroanatomical Correlates of Recognizing Face Expressions in Mild Stages of Alzheimer’s Disease
Source: PLoS One. 2015 Dec 16;10(12):e0143586. doi: 10.1371/journal.pone.0143586 (PMC4684414; doi:10.1371/journal.pone.0143586)
Supplement: S1 Table — R: right, L: left. IR: impaired recognition. r: Pearson correlation coefficient. Green = negative correlations, red = positive correlations. *p<0.05, **p<10–2, ***p<10–3. Literature: Region hypothesized to be involved in task a priori. These correlations were not corrected for multiple comparisons; compare with Table 2 in the main article. (DOCX) [file pone.0143586.s002.docx]

| **Emotion** | **Region** | **Side** | **Intensity** | **r** | **Literature** |
| --- | --- | --- | --- | --- | --- |
| **Neutral** | *Lingual gyrus* | R  L | **-** | -0.37 *  -0.33 * |  |
|  | *Anterior orbital gyrus* | R | **-** | 0.38 * |  |
| **Anger** | *Putamen* | R  L | 100%  100% | -0.37 *  -0.37 * | ✓ |
|  | *Gyri parahippocampalis et ambiens* | R | 40% | -0.34 * | ✓ |
|  | *Precentral gyrus* | L | 100% | -0.39 * | ✓ |
|  | *Lingual gyrus* | R  L | 40%  100%  Average  **IR**  100% | -0.33 *  -0.37 *  -0.42 **  -0.42 **  -0.33 * | Occipital lobe |
|  | *Cuneus* | R | 100% | -0.34 * | Prestriate |
|  | *Posterior orbital gyrus* | R | Average  **IR** | -0.31 *  -0.32 * | Anterior and medial OFC |
|  | *Straight gyrus, gyrus rectus* | R | 60% | 0.34 * |  |
|  | *Postcentral gyrus* | L | 60%  100% | -0.32 *  -0.43 ** |  |
|  | *Superior parietal gyrus* | R  L | 60%  100%  60% | -0.42 **  -0.34 *  -0.32 * |  |
| **Fear** | *Amygdala* | R  L | 60%  100%  Average  **IR**  100% | -0.40 *  -0.47 ***  -0.36 **  -0.36 **  -0.40 ** | ✓ |
|  | *Hippocampus* | R  L | 100%  100% | -0.42 **  -0.36 * | ✓ |
|  | *Pallidium. globus pallidus* | R | 80% | -0.33 * | ✓ |
|  | *Lateral orbital gyrus* | R | 20% | 0.37 * | ✓ |
|  | *Anterior cingulate gyrus* | L | 60% | 0.33 * | ✓ |
|  | *Cuneus* | R | 100% | -0.38 * | Occipital lobe |
|  | *Pre-subgenual frontal cortex* | L | 100% | 0.41 * |  |
| **Disgust** | *Pallidum, globus pallidus* | R  L | 20%  20%  **40%=IR** | -0.39 *  -0.49 **  -0.38 * | ✓ |
|  | *Posterior superior temporal gyrus* | L | 20% | -0.33 * | ✓ |
|  | *Anterior cingulate gyrus* | L | **40%=IR**  80% | 0.42 **  0.45 ** | ✓ |
|  | *Medial orbital gyrus* | R | **40%=IR** | 0.33 * | ✓ |
|  | *Subgenual frontal cortex* | R | 60% | 0.39 * |  |
|  | *Corpus callosum* | - | 20% | -0.35 * |  |
| **Happiness** | *Lateral occipitotemporal gyrus,*  *gyrus fusiformis* | R  L | 20%  40%  **IR**  40%  60%  Average  **IR** | -0.33 *  -0.34 *  -0.38 *  -0.33 *  -0.45 **  -0.47 **  -0.51 ** | ✓ |
|  | *Insula* | L | 40% | -0.38 * | ✓ |
|  | *Occipital lobe* | R | 100% | 0.35 * | ✓ |
|  | *Lingual gyrus* | L | 100% | 0.36 * | ✓ |
|  | *Posterior cingulate gyrus* | R | 20% | 0.41 ** | Anterior cingulate g. |
|  | *Subcallosal area* | L | 80%  100%  Average | -0.39 *  -0.39 *  -0.35 * |  |
|  | *Superior parietal gyrus* | R  L | 40%  100%  100% | -0.34 *  0.32 *  0.38 * |  |
|  | *Parietal lobe* | L | 100% | 0.37 * |  |
| **Total of the**  **4 emotions** | *Amygdala* | R  L | - | -0.39 *  -0.33 * | ✓ |
|  | *Hippocampus* | L | - | -0.34 * | ✓ |
|  | *Medial orbital gyrus* | R | - | 0.13 * | ✓ |
|  | *Anterior cingulate gyrus* | L | - | 0.35 * | ✓ |

**Supplementary Table: *Correlations between emotion recognition performance and regional volumes in the patient group***

*R*: right, *L*: left. *IR*: impaired recognition. *r*: Pearson correlation coefficient. Green=negative correlations, red=positive correlations. *p<0.05, **p<10^-2^, ***p<10^-3^. Literature: Region *a priori* hypothesised to be involved in task. These correlations were not corrected for multiple comparisons; compare with Table 2 in the main article.
